# Analysing horizontal equity in enrolment in Disease Management Programmes for coronary heart disease in Germany 2008–2010

**DOI:** 10.1186/s12939-015-0155-1

**Published:** 2015-03-10

**Authors:** Kayvan Bozorgmehr, Miguel San Sebastian, Hermann Brenner, Oliver Razum, Werner Maier, Kai-Uwe Saum, Bernd Holleczek, Antje Miksch, Joachim Szecsenyi

**Affiliations:** Department of General Practice & Health Services Research, University Hospital Heidelberg, Heidelberg, Germany; Division of Epidemiology and Global Health, Department of Public Health and Clinical Medicine, Umeå University, Umeå, Sweden; Division of Clinical Epidemiology and Aging Research, German Cancer Research Center (DKFZ), Heidelberg, Germany; Department of Epidemiology & International Public Health, School of Public Health, Bielefeld University, Bielefeld, Germany; Institute of Health Economics and Health Care Management, Helmholtz Zentrum München - German Research Center for Environmental Health (GmbH), Neuherberg, Germany; Saarland Cancer Registry, Saarbrücken, Germany

**Keywords:** Equity, Health services, Horizontal inequity index, Concentration index, Regional deprivation, Coronary heart disease

## Abstract

**Background:**

Disease Management Programmes (DMPs) have been introduced in Germany ten years ago with the aim to improve effectiveness and equity of care, but little is known about the degree to which enrolment in the programme meets the principles of equity in health care. We aimed to analyse horizontal equity in DMP enrolment among patients with coronary heart disease (CHD).

**Methods:**

Cross-sectional analysis of horizontal inequities in physician-reported enrolment in the DMP for CHD in a large population-based cohort-study in Germany (2008–2010). We calculated horizontal inequity indices (HII) and their 95% confidence intervals [95%CI] for predicted need-standardised DMP enrolment across two measures of socio-economic status (SES) (educational attainment, regional deprivation) stratified by sex. Need-standardised DMP enrolment was predicted in multi-level logistic regression models.

**Results:**

Among N = 1,280 individuals aged 55–84 years and diagnosed with CHD, DMP enrolment rates were 22.2% (women) and 35.0% (men). Education-related inequities in need-standardised DMP enrolment favoured groups with lower education, but HII estimates were not significant. Deprivation-related inequities among women significantly favoured groups with higher SES (HII = 0.086 [0.007 ; 0.165]. No such deprivation-related inequities were seen among men (HII = 0.014 [−0.048 ; 0.077]). Deprivation-related inequities across the whole population favoured groups with higher SES (HII estimates not significant).

**Conclusion:**

Need-standardised DMP enrolment was fairly equitable across educational levels. Deprivation-related inequities in DMP enrolment favoured women living in less deprived areas relative to those living in areas with higher deprivation. Further research is needed to gain a better understanding of the mechanisms that contribute to deprivation-related horizontal inequities in DMP enrolment among women.

**Electronic supplementary material:**

The online version of this article (doi:10.1186/s12939-015-0155-1) contains supplementary material, which is available to authorized users.

## Introduction

Coronary heart disease (CHD) is the leading cause of death and an important cause of morbidity world-wide [1,2] and in Germany [3]. As part of a nation-wide attempt to restructure health care for CHD and other chronic diseases, Disease Management Programmes (DMPs) were introduced between 2002 and 2005 into the German statutory health insurance system (SHI). Their overall goals are to reduce morbidity, improve survival and quality of life of enrolled patients [4]. To this end, DMPs aim to improve quality of care for the chronically ill by aligning service delivery with evidence-based guidelines and with a ‘managed care approach’ that has been shown to increase effectiveness of care [5]. Another major rationale of the DMPs was to provide financial incentives for purchasers (i.e. sickness funds) and health service providers to care for the chronically ill. This would (so the assumption) reduce “cream-skimming”, and increase equity in the system [6] by allocating resources to sickness funds and providers who care for patients with higher needs.

In contrast to managed care approaches in other countries [7], German DMPs are tightly linked to sickness funds [6], and their core content (guideline-oriented care, quality indicators, coordinated transfer between different levels of care, recall for patients) is defined by a national expert group whose recommendations are binding for providers, while allowing for smaller differences among different contracting partners with respect to implementation [5]. The first DMPs for CHD (DMP-CHD) were introduced in 2003. Ten years later, more than 1.7 million patients across Germany were enrolled in about 1,700 accredited DMPs for CHD [8]. Participation in DMPs is voluntary for patients, but physicians have the mandate to enrol only “active patients” with respect to their therapy who can potentially benefit from the programme [4]. Evidence suggests that patients enrolled in the DMP-CHD do not benefit in terms of lower mortality [9], but receive a better quality of care [10].

Previous research in the context of DMPs has mainly assessed programme effectiveness [9,11,12]. Despite concerns about selective enrolment favouring patients with higher socio-economic status (SES) [13], and despite considerably higher resources allocated to patients enrolled in the DMPs [14], no studies have yet analysed equity in DMP enrolment. The concern with selective enrolment is not only important for conclusions on programme effectiveness [11,13]. Inequitable enrolment in DMPs, i.e. selective enrolment based on socio-economic differences, could (theoretically) lead to intervention-generated inequalities [15], provided that the programme (or elements thereof) are effective in achieving their goals. We therefore aimed to assess if the principles of horizontal equity (equal treatment for equal need regardless of socio-economic factors [16]) are met in the context of the DMP-CHD.

## Methods

### Design and study population

We conducted a cross-sectional analysis using data of the “Epidemiological Study for the Prevention, Early Diagnosis and Optimal Treatment of Chronic Diseases in an Elderly Population” (ESTHER). This prospective (ongoing) cohort study includes non-institutionalized people from the general population living in the German federal state of Saarland, who were recruited by their general practitioners (GPs) during a general health check-up between 2000 and 2002 (t0). Baseline recruitment (t0) occurred before DMPs were introduced [6].

A total of 9,949 out of 12,000 invited individuals, aged 50 to 75 years, agreed to participate in the cohort. This sample is representative for the population of Saarland in the respective age range [17]. Numerous GP-reported and patient-reported measures were captured in the GP-practice by questionnaires at baseline, and by postal questionnaires after two (t1), five (t2), eight (t3) and 11 (t4) years of follow-up [17-19]. For the purpose of this study, we focused on the population at t3 (2008–2010) since information on DMP enrolment was not captured in previous follow-ups. At t3, the cohort consisted of N = 7,012 survivors that were still physically and mentally able to participate (response rate 80.9%).

### Inclusion and exclusion criteria

**Figure 1:**
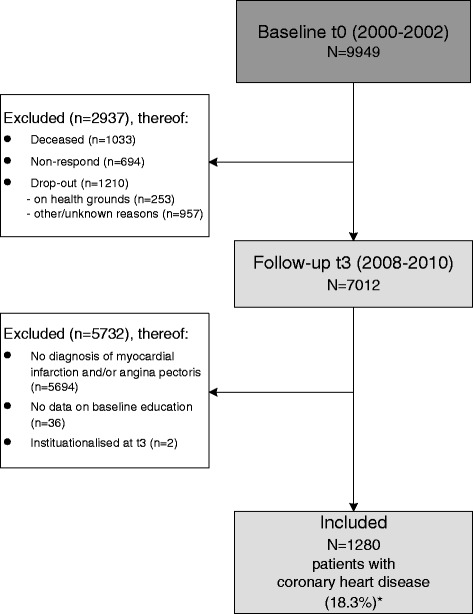
**Flowchart of patient selection process according to inclusion/exclusion criteria.** *Proportion refers to population at t3 (N = 7,012).

We a priori excluded participants who deceased, did not respond or dropped out since t0 on health grounds or other and unknown reasons. Further criteria for exclusion were defined as not having a GP-reported diagnosis of myocardial infarction (MI) and/or angina pectoris (AP) between t0 and t3, missing data on baseline SES, and being institutionalised at t3 (Figure 1).

### Measurement of horizontal (in-)equity in DMP enrolment and statistical analysis

We measured horizontal (in-)equity by estimating the concentration index and 95% confidence intervals [95%CI] for need-standardised enrolment, which has been referred to as the horizontal inequity index (*HII*) ranging from −1 to +1 [20]. The *HII*, a measure of (in-)equity in health care use, can be obtained by a direct or an indirect standardisation method [20,21] in order to account for (legitimate) socio-economic differences in enrolment based on differences in need. As pointed out by O’Donnell et al., this procedure rests on the assumption that once observable need indicators have been controlled for, any residual variation in utilization is attributable to non-need factors [21].

A positive *HII* indicates a higher share of health care use by groups with higher SES than their share of need, i.e. horizontal inequities favouring the better-off. A negative value of *HII* represents horizontal inequities favouring groups with lower SES given their share of need. The *HII* is zero if health care use is equitably distributed across the socio-economic groups [22], i.e. if there is no inequality in the share of health care use given the share of need between respective socio-economic groups.

#### Need variables

Enrolment in the DMP-CHD is voluntary. Patients who are diagnosed with CHD are eligible, provided that their GP offers DMP-CHD and considers the patient to be “active” and “likely to benefit from the programme”. The DMP-CHD consists of regular follow-up visits, need-based pro-active therapy, life-style counselling, psychosocial counselling, and standardised reference pathways to specialists and other levels of care. The programme goals are to reduce mortality and cardiovascular morbidity, prevent recurrent cardiovascular events and heart failure, and increase quality of life [4]. In practice, patients with severe co-morbidities, or suffering from life-limiting conditions, or of very high age might be judged to be “not likely” to benefit from the programme in terms of the goals, as might those with healthy lifestyles and well-controlled risk factors. Therefore, our need variables should ideally account for potential differences in enrolment that are related to differences in co-morbidity and CHD risk factors.

To this end, we used age (three categories), sex (male/female), objective (Cumulative Illness Rating Scale for Geriatrics Severity Index, CIRS-G [23]) and subjective (self-rated health as dummy: “excellent, very good” vs. “fair, bad, very bad”) measures of morbidity simultaneously as need variables.

The CIRS-G is a comprehensive (physician-rated) assessment of 14 organ systems, reflecting not only the presence, but also the severity of any physical (co-)morbidity. Self-rated health (SRH) in turn is a global measure with high prognostic validity for morbidity and mortality, and is used in this study to reflect the psychosocial dimension of need for continuous and pro-active care as provided by the DMP.

Although further variables could theoretically be considered as reflecting “need to enrol” (e.g. smoking, body mass index etc.), we limited the analysis to the above-mentioned variables mainly because previous analyses in the cohort (unpublished) showed that there are no systematic differences in DMP-CHD enrolment based on life-style factors.

Following the indirect standardisation method with non-linear models [20,21], we obtained need-expected enrolment in the DMP-CHD (dummy) in multi-level logistic regression models (cross-classified models), which contained all need variables (age, sex, CIRS-G, SRH) and accounted for the simultaneous clustering of patients in both municipalities and in GP-practices. Details on the model specification are provided in the supplementary file along with regression coefficients, standard errors, and variance parameters obtained from the multi-level regression models (Additional file 1).

#### Control variables

Non-need variables included in the analysis were the number of social contacts, i.e. family members/friends whom participants can count upon or discuss problems with (included as a proxy of loneliness in the elderly), and an immigration background (dummy), defined as having (i) a foreign nationality or (ii) a German nationality and a place of birth outside of Germany. The number of social contacts was initially operationalised as a variable with three categories (“0-1”, “2-4”, “5 and more”). It was collapsed to a binary variable (“0-1” vs. “2 and more”) for inclusion in the regression model to avoid collinearity. Non-need variables were set to their means in obtaining the predictions [21].

#### Inequality in need-standardised DMP enrolment

The predicted values obtained from the multi-level logistic regression models can be interpreted as need-*expected* enrolment, adjusted for non-need variables. Random effects (at the level of the cross-classification between municipalities and GP-practices) were set to zero in predicting need-expected enrolment. As explained in details elsewhere [20,21], need-*standardised* enrolment was obtained by subtracting need-*expected* enrolment from the observed *actual* enrolment.

In a next step, we calculated the concentration index for need-standardised DMP-CHD enrolment (*HII*) to assess inequalities across two measures of SES stratified by sex. First, the highest educational attainment at baseline, defined as an ordinal, individual-level SES variable with three levels contrasting low (level I - no formal degree or at least 9 years of schooling, *Hauptschule*), medium (level II – at least 10 years of schooling qualifying for professional training, *Realschule/Mittlere Reife*) and high (level III – at least 12 or 13 years of schooling qualifying for university entrance, *Fachhochschulreife*/*Abitur*).

Second, small-area regional deprivation of patients’ residential area using the German Index of Multiple Deprivation (GIMD) [24]. The GIMD includes seven domains of area deprivation: income, employment, education, municipal revenue, social capital, environment, and security [25]. Patients were assigned a distinct value of regional deprivation at municipality level by linking their ZIP codes (at t3) to one of 52 municipalities in Saarland. Municipalities were grouped into quintiles (Q1: lowest SES/highest deprivation – Q5: highest SES/lowest deprivation) in relation to all 9,620 municipalities in Germany.

When predicting the need-standardised enrolment for analysis across regional deprivation as SES indicator, we included educational attainment as non-need variable in the multi-level logistic regression model (Additional file 1). The education variable with three categories was collapsed to a binary variable (“level I” vs. “level II + II”) to avoid collinearity. The rationale for including education as non-need variable was to assess the inequity across regional deprivation while controlling for the effects of individual-level education and to reduce the possibility of omitted variable bias.

We plotted concentration curves for the *HIIs* across each SES indicator to illustrate the distribution of DMP-CHD enrolment across social groups. For descriptive purposes, DMP enrolment rates stratified by sex and socio-economic group were calculated in addition to sex-stratified absolute frequencies/proportions for all variables used in this study.

All analyses were performed using Stata® 12.1. The Distributive Analysis Stata Package (DASP) [26] with the commands “*igini*” and “*clorenz*” was used to calculate the concentration indices of need-standardised enrolment (*HII*) including standard errors and 95% CIs, and to plot the concentration curves respectively. Details on the calculation of standard errors by the DASP command “*igini*” (following personal communication with the authors of the package) are provided in Additional file 2. To assess the robustness of inferences we additionally estimated CIs at the 90% level for all *HII*s.

#### Missing data

Patients without ZIP codes (n = 14) or without GP-identifier (n = 25) were not included in the regression analysis. Missing data in covariables were treated as missing at random and a complete case analysis was performed.

## Results

### Descriptive results

The lifetime prevalence of CHD in the cohort at t3 (N = 7,012) was 18.8% (n = 1,318). Of these, N = 1,280 (37.3% women) fulfilled all inclusion criteria (Figure 1). The lifetime prevalence of MI in this sub-sample was lower among female (26.3%) compared to male participants (44.1%), while the prevalence of AP (94.3%) in the sub-sample was fairly balanced. Mean age was 72.3 years (SD 6.2). More than three-quarters of the sub-sample with CHD had a low educational level and one quarter lived in municipalities categorised as most deprived (Q1, lowest SES) (Table 1).

**Table 1:** **Descriptive characteristics of included participants of the ESTHER study with coronary heart disease at the 8-year follow-up (2008–2010) (N = 1,280)**

		**Female**	**Male**	**Total**
		**Freq. (col %)**		
Age group	55-64	33 (6.9)	132 (16.5)	165 (12.9)
	65-74	241 (50.4)	386 (48.1)	627 (49)
	75-84	204 (42.7)	284 (35.4)	488 (38.1)
	N (%)	478 (100)	802 (100)	1280 (100)
*Socio-economic status (SES)*				
Highest educational attainment^*^	Level I (lowest)	402 (84.1)	591 (73.7)	993 (77.6)
	Level II	56 (11.7)	84 (10.5)	140 (10.9)
	Level III (highest)	20 (4.2)	127 (15.8)	147 (11.5)
	N (%)	478 (100)	802 (100)	1280 (100)
Regional deprivation of patients’ residential areas (GIMD)	Q1 (lowest SES/highest deprivation)	126 (26.53)	194 (24.53)	320 (25.28)
	Q2	164 (34.53)	247 (31.23)	411 (32.46)
	Q3	89 (18.74)	147 (18.58)	236 (18.64)
	Q4	64 (13.47)	142 (17.95)	206 (16.27)
	Q5 (highest SES/lowest deprivation)	32 (6.74)	61 (7.71)	93 (7.35)
	N (%)	475 (100)	791 (100)	1266 (100)
*Lifetime prevalence of physician-reported CHD defining morbidities/index diseases*				
Myocardial infarction	Yes	118 (26.3)	346 (44.1)	464 (37.6)
	N (%)	448 (100)	785 (100)	1233 (100)
Angina pectoris	Yes	459 (96)	745 (93.2)	1204 (94.3)
	N (%)	478 (100)	799 (100)	1277 (100)
*Need-variables*				
Cumulative Illness Rating Scale for Geriatrics (CIRS-G) severity index^**^	M (SD)	1.60 (0.43)	1.61 (0.45)	1.61 (0.44)
	N	394	663	1057
Self-rated health	Excellent/very good	182 (53.4)	397 (62.2)	579 (59.1)
	Fair/bad/very bad	159 (46.6)	241 (37.8)	400 (40.9)
	N (%)	341 (100)	638 (100)	979 (100)
*DMP utilization (physician-reported)*				
Enrolled in DMP	No	372 (77.8)	521 (65)	893 (69.8)
	Yes	106 (22.2)	281 (35)	387 (30.2)
	N (%)	478 (100)	802 (100)	1280 (100)
Duration of enrolment in DMP	low (0.5-3yrs)	61 (64.2)	154 (60.4)	215 (61.4)
	high (4-7yrs)	34 (35.8)	101 (39.6)	135 (38.6)
	N (%)	95 (100)	255 (100)	350 (100)
*Non-need factors*				
Immigration background^***^	Yes	45 (9.5)	60 (7.5)	105 (8.2)
	N (%)	475 (100)	798 (100)	1273 (100)
Social contacts: family members/friends whom participants can count upon/discuss problems with	0-1	48 (14.2)	93 (14.7)	141 (14.5)
	2-4	184 (54.4)	331 (52.2)	515 (53)
	5-10 and more	106 (31.4)	210 (33.1)	316 (32.5)
	N (%)	338 (100)	634 (100)	972 (100)

All data refer to the 8-year follow-up phase (t3: 2008–2010) except baseline data taken from t0 (education, sex, immigration background). **M**: arithmetic mean. **SD**: standard deviation. **Freq**.: absolute frequency. **Col%:** column percent. ^*^ **Highest educational attainment**: Level I: no degree or minimum of nine years of education qualifying for professional training (Hauptschule). Level II: minimum of 10–11 years of education qualifying for professional training (Realschule/Mittlere Reife). Level III: minimum of 12–13 years of education qualifying for university entrance (Fachhochschulreife/Abitur). ***CIRS-G: Cumulative Illness Ranking Scale for Geriatrics, severity index calculated as CIRS-G score divided by the number of endorsed CIRS-G categories (physician-reported). ^***^ **Immigration background**: defined as having (i) a foreign nationality or (ii) a German nationality and a place of birth outside of Germany. **Q1-Q5**: Quintiles of the German Index of Multiple Deprivation (GIMD).

**Figure 2:**
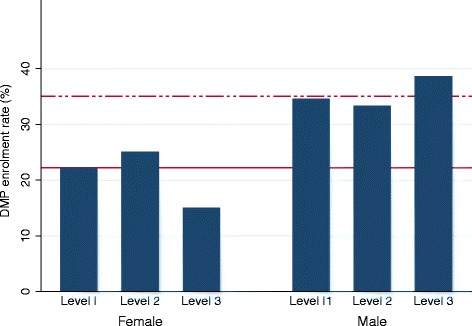
**Rate of enrolment in the Disease Management Programme for coronary heart disease by highest educational attainment and sex.** *Highest educational attainment: Level I: no degree or minimum of nine years of education qualifying for professional training (Hauptschule). Level II: minimum of 10–11 years of education qualifying for professional training (Realschule/Mittlere Reife). Level III: minimum of 12–13 years of education qualifying for university entrance (Fachhochschulreife/Abitur). DMP: Disease Management Programme for coronary heart disease. Solid horizontal reference line: mean enrolment rate among female. Dashed horizontal reference line: mean enrolment rate among male.

**Figure 3:**
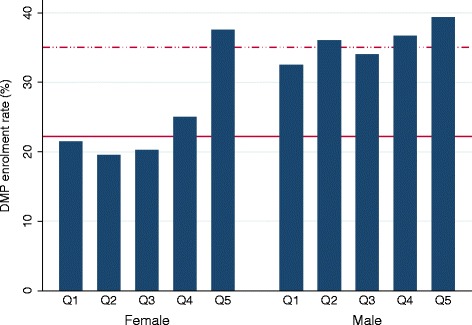
**Rate of enrolment in the Disease Management Programme for coronary heart disease by regional deprivation and sex. Q1-Q5**: Quintiles of the German Index of Multiple Deprivation (GIMD), where Q1 indicates lowest socio-economic status (highest deprivation) and Q5 indicates highest socio-economic status (lowest deprivation). DMP: Disease Management Programme for coronary heart disease. Solid horizontal reference line: mean enrolment rate among female. Dashed horizontal reference line: mean enrolment rate among male.

The majority (72.6%) of patients enrolled in the DMP-CHD (N = 387) were male. The overall DMP enrolment rates were 22.2% among women (N = 478) and 35.0% among men (N = 802). The observed actual DMP-CHD enrolment rates by education for men and women did not follow a clear pattern (Figure 2), while the observed actual enrolment rates by regional deprivation indicated the existence of a social gradient, particularly among women (Figure 3). Absolute frequencies and rates of enrolment (or more precisely: the prevalence of enrolment) by social status and sex are provided in detail in Additional file 1.

The proportion of women in lower age-groups (55–64) was 10 percentage-points lower compared to men, while the proportion of women was higher (7 percentage-points) in the highest age-group (75–84). The severity of (co-)morbidities was fairly balanced between both sexes, while a higher share of women (8 percentage-points more than men) rated their health status as “fair, bad, very bad” (Table 1).

### Horizontal inequity indices

#### Education-related inequities

**Figure 4:**
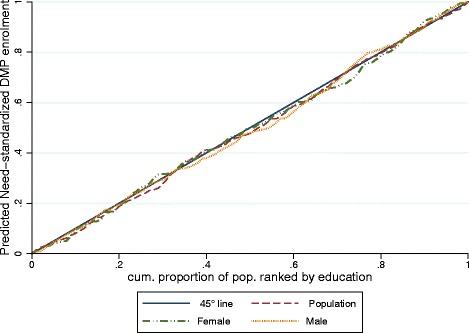
**Concentration curve of the need-standardised enrolment in the Disease Management Programme for coronary heart disease by highest educational attainment and sex.** 45° line: indicates absence of horizontal inequities. A curve below (above) the 45° line indicates horizontal inequity favoring groups with higher (lower) educational attainment. Need variables used for standardization: Cumulative Illness Rating Scale severity index, age, sex and self-rated health. The predicted need-standardised enrolment is adjusted for the non-need factors immigration background and social contacts. Prediction based on sample size of N = 720 individuals, derived from a (cross-classified) multi-level logistic regression model. X-axis: cumulative proportion of population ranked by highest educational attainment.

The *HIIs* across educational attainment were small, slightly concentrated among groups with lower education and not significant at the 95% level both across the whole population (*HII* = −0.024 [−0.072; 0.024] ) and in sex-stratified analyses. All estimates related to educational attainment as rank variable remained non-significant at the 90% level (Table 2). The concentration curves of the population-wide and sex-stratified *HIIs* for DMP enrolment across education mainly lay on the diagonal (*HII* = 0) indicating absence of inequities (Figure 4).

**Table 2:** **Horizontal inequity indices and 95% confidence intervals for enrolment in the Disease Management Program for coronary heart disease across highest educational attainment and regional deprivation by sex**

	**Female**	**Male**	**Population**	
**SES-variable**	**Horizontal inequity index -** ***HII*** ***(SE)*** **[95% CI]**			**N**
Highest educational attainment^*^	**−0.012**	**−0.04**	**−0.024**	720
	*(0.040)*	*(0.032)*	*(0.025)*	
	[−0.091; 0.067]	[−0.010; 0.022]	[−0.072; 0.024]	
Regional deprivation of patients’ residential areas (GIMD)^**^	**0.086**	**0.014**	**0.036**	720
	*(0.040)*	*(0.032)*	*(0.025)*	
	[0.007; 0.165]	[−0.048; 0.077]	[−0.012; 0.085]	

*HII*: Concentration index of need-standardized enrolment. Need variables used for standardization: Cumulative Illness Rating Scale severity index, age, sex and self-rated health. *HII is adjusted for the non-need factors immigration background and social contacts. **HII is adjusted for the individual-level non-need factors educational attainment (dummy: Level I vs. Level II + III), immigration background (dummy: yes/no) and social contacts (dummy: 0–1 contacts vs. 2 or more). N = sample size on which the need-standardized prediction is based (complete case analysis). Differences to N = 1,280 due to missing data in covariables and/or municipality ID and/or GP-practice ID. GIMD: German Index of Multiple Deprivation. 90% CIs for HII estimates across highest educational attainment: Female [−0.078; 0.054], Male [−0.092; 0.012], Population [−0.065; 0.017]. 90% CIs for HII estimates across GIMD: Female [0.012; 0.152], Male [−0.038; 0.066], Population [−0.005; 0.080].

#### Regional deprivation-related inequities

Compared to the education-related inequities, horizontal inequities across regional deprivation showed a positive sign and thus favoured groups with higher SES. Deprivation-related inequities among women were significantly different from zero (*HII* = 0.086 [0.007; 0.165]), whereas no such differences were seen among men (*HII* = 0.014 [−0.048; 0.077]). Deprivation-related inequities were not significant across the whole population at the 95% level (HII = 0.036 [−0.012; 0.085]), but were marginally significant at the 90% level (Table 2).

**Figure 5:**
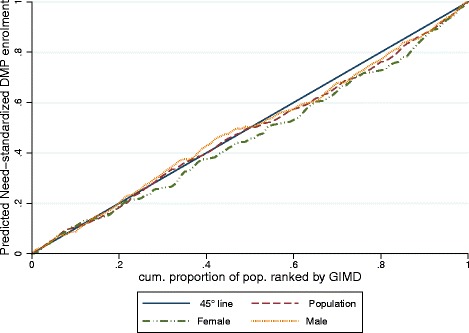
**Concentration curve of the need-standardised enrolment in the Disease Management Programme for coronary heart disease by regional deprivation and sex.** 45° line: indicates absence of horizontal inequities. A curve below (above) the 45° line indicates horizontal inequity favoring groups with higher (lower) socio-economic status measured by the German Index of Multiple Deprivation (GIMD). Need variables used for standardization: Cumulative Illness Rating Scale severity index, age, sex and self-rated health. The predicted need-standardised enrolment is adjusted for the non-need factors highest educational attainment, immigration background and social contacts. Prediction based on sample size of N = 720 individuals, derived from a (cross-classified) multi-level logistic regression model. X-axis: cumulative proportion of population ranked by quintiles of the German Index of Multiple Deprivation (GIMD).

The concentration curve for need-standardized DMP enrolment across regional deprivation lay below the diagonal, particularly for women (Figure 5), showing that the enrolment was inequitably distributed given their share of need, favouring women living in municipalities with higher SES. The population-wide and male-specific curve lay below the diagonal mainly for the upper 50% of the population, indicating that inequities were more relevant across medium and higher SES categories (i.e. lower deprivation), while the (population-wide and male-specific) distribution was fairly equitable for the 50% of the population with lowest SES (i.e. highest deprivation).

## Discussion

This is the first study analysing horizontal equity in DMP enrolment among elderly people with CHD in Germany, taking into account individual need by means of *both* objective *and* subjective measurements while controlling for *both* non-need variables *and* the (simultaneous) clustering of patients in municipalities and GP-practices.

We found evidence for horizontal inequities favouring women living in less deprived municipalities relative to those living in municipalities with higher deprivation. The enrolment of women in the DMP-CHD did not correspond with their level of need, adjusted for differences in educational attainment and other non-need variables (social contacts, immigration background).

Our findings provide evidence that the principle of equal access or treatment for equal need is not fulfilled with respect to the systematic differences depending on small-area regional deprivation. Women in socially deprived areas are more disadvantaged than male patients with the same need. The degree to which “potential access” to the DMP-CHD (guaranteed by the SHI system) is converted into “realised access” [27] appears to be affected by regional deprivation and the (gender-specific) mechanisms underlying this association deserve further investigation.

A very positive finding is that DMP enrolment widely meets the principles of equity in health care as far as individual educational attainment (interpreted as an indicator for SES) is concerned. In this respect, primary care services in the federal state of Saarland produced an equitable distribution of health care services and resources.

This finding is consistent with two studies that assessed the possibility of selection effects in enrolment in DMP-CHD using self-reported [9] and physician-reported [10] enrolment status in an other German federal state. These studies [9,10] tested the hypothesis that there are no differences between enrolled and non-enrolled patients using a wide range of factors, including individual-level educational attainment. However, the comparability is limited because these studies [9,10] (and other studies focusing on the DMP Diabetes [12,13,28]) primarily assessed average “differences” across educational groups, and did not explicitly focus on equity or inequity in enrolment. This means that differences in need or morbidity were not taken into account when analysing potential education-related differences in enrolment.

### Strengths and limitations

The major strength of our study is that we assessed equity in GP-reported DMP-CHD enrolment using individual-level data from a population-based cohort study. This allowed the use of appropriate measures of need, while controlling for relevant non-need factors. It might be argued that no need-standardisation would be necessary if the aim of DMPs is to include *everyone* diagnosed with a CHD. Reaching this ‘ideal’ might have been an intrinsic motivation to introduce DMPs, but insights from qualitative studies show that implementation of DMPs at GP-practice level is challenged by a lack of time and human resources, so that the programme is in reality not offered to everyone with a relevant condition [29]. Consideration of some aspect of need thus remains crucial when trying to assess equity in enrolment, given that *implicit* judgements on eligibility and need are made by physicians in light of limited resources.

We used the CIRS-G severity index and SRH to operationalise need among CHD patients for a structured and intensified treatment of their condition in the scope of the DMP-CHD. We believe the variables are a valid reflection of need for enrolment in this context, but it is important to note that the legal regulation [4] does not explicitly formulate specific need criteria. It only combines formal eligibility criteria (CHD diagnosis) with vaguely defined criteria amenable to subjective judgements by physicians. Studies that operationalise need for treatment in the DMP-CHD differently might thus yield different results.

Previous studies on DMPs in Germany were not able to assess the possibility of socio-economic selection processes between participants and non-participants due to the limited availability of specific information on SES and/or lack of control groups [11-13,30]. It is also not possible to evaluate equity in enrolment by means of routine or claims data because of the lack of control groups (i.e. data on non-participants) and rudimentary information on individual covariables. The availability of individual-level educational attainment for both enrolled and non-enrolled patients, and the possibility of linking patients to area-based measures of SES (GIMD) allowed a comprehensive assessment of equity aspects in our study. This study thus adds to previous studies on selection effects in DMP enrolment [9,10,12,13,28] in using a comprehensive measure of SES such as the GIMD in addition to educational attainment. If previous studies used SES indicators to assess selective enrolment, then they relied on educational attainment alone, which is an arguably imperfect indicator of SES in elderly populations [31]. The socio-economic variables were sometimes even treated as covariables to be adjusted for in the analysis [10,12]. In contrast to the above studies, this analysis focused explicitly on the socio-economic distribution of need-standardised propensity of DMP enrolment.

Another strength is the use of concentration indices (*HII*) to estimate (in-)equity in enrolment, which – to the best of our knowledge – has not been done in the context of health care use in Germany previously. This measure takes into account inequalities across the whole distribution of socio-economic groups, in contrast to conventional epidemiological methods (e.g. measures of association such as frequency ratios or odds ratios) which establish the presence of inequalities by comparing two or more socioeconomic groups without taking into account how individuals are distributed in each SES category [32]. The use of the concentration index is hence recommended for comparisons between populations (e.g. from other federal states of Germany with different sizes of respective socio-economic groups), and over time [32] where differences in sizes of socio-economic groups might affect estimates derived from conventional epidemiological methods. Our estimates of (in-)equity in enrolment of the DMP-CHD can thus be used as baseline for monitoring change in (in-)equity over time e.g. in the ongoing ESTHER study or in other cohort studies with comparable data basis [9,10]. As such, our study contributes to attempts for improving the evaluation of German Disease Management Programmes which has been referred to as insufficient elsewhere [33].

Several methodological issues [34-36] should be noted in this context. First, our study analyses inequity in a binary outcome. As O’Donnell et al. point out, the concentration index is an appropriate measure of socio-economic health (care) inequality when health care is measured on a ratio scale with nonnegative values [37]. In line with common approaches [34,38,39], we have therefore transformed the binary outcome to a ratio scale in using predicted probabilities as outcome.

The fact that predicted probabilities are bounded (i.e. have a range between zero and 100%) creates two other challenges: a) The range of the concentration index is linked to the mean of the variable [34,36], in our case the mean prevalence of DMP enrolment. b) The “mirror principle” [40] is not fulfilled, which means that inequalities may vary depending on whether we measure inequality in attainment (e.g. DMP enrolment) or in shortfall (e.g. non-enrolment in DMPs) [38]. In other words: inequalities may vary depending on which attribute is coded 0 and 1 [36].

To circumvent challenge (a), a normalisation to the mean of the outcome has been proposed [41]. The underlying value judgements of the normalised concentration index, however, lead to the fact that different pro-rich distributions yield the same value of inequality [34]. In line with suggested options to deal with the “dilemma” [36] faced by the analyst, we decided to stick with the (unnormalized) concentration index but “be aware of the limitations of the analysis” (which may not arise unless one attempts to measure inequities in enrolment between federal states or over time where the average rate of enrolment varies strongly).

As for challenge (b), several options have been suggested to achieve the desired mirror principle [38]. Others argue that the issue is of less concern if there is a convention on what to code “1” [36]. Although there is no explicit convention in our case, all studies on DMPs in Germany have yet analysed “enrolment”. We therefore think that the mirror issue is less of a concern if future analyses on equity in DMP enrolment stick to the analysis of “enrolment” as outcome (instead of “non-enrolment”) when using concentration indices.

Another methodological issue worth mentioning is that we used the GIMD as ordinal variable in quintiles as is common practice in analyses using area-based deprivation measures [42]. Although the literature on income-related health inequality usually uses the socio-economic ranking variable on a continuous scale, it is important to note that an ordinal scale is sufficient [38,43-45]. There are also examples where continuous measures of SES are transformed to an ordinal scale for use as ranking variable in order to calculate the concentration index based on quintiles [46], as done in our analysis with the GIMD.

Our findings regarding equity or inequity in DMP-CHD enrolment can be generalised to Saarland but not necessarily to Germany as a whole. This is mainly due to (i) the intra- and inter-regional heterogeneity to which DMPs are implemented across each federal state, and (ii) the non-significance or marginal statistical significance of some of the *HII* estimates in our study (Table 2). It should also be noted that our results provide insights into equity distributions in the respective time period, but that the situation might have changed since data for this study was collected. This underlines the importance of monitoring of equity aspects in health services utilisation on a routine basis.

Our findings are limited by the cross-sectional nature of the study and the possibility of attrition bias, because the DMP enrolment status of those who deceased or dropped out before t3 was not known. Furthermore, we had information only on current enrolment, which means that individuals who might have been enrolled before t3 but eventually opted out of the DMP until t3, were categorised as “non enrolled”. In performing a complete case analysis, we excluded 560 individuals from the calculation of *HII*s, which might have affected our results.

## Conclusion

Need-standardised DMP enrolment in the federal state of Saarland was fairly equitable across educational levels. The principles of horizontal equity, however, were not fulfilled for CHD patients - particularly women - since systematic differences existed between those living in deprived and less deprived municipalities. Deprivation-related inequities in DMP enrolment significantly favoured women living in less deprived areas relative to those in areas with higher deprivation. Further research (in form of decomposition analyses or qualitative approaches) is needed to gain a better understanding of the mechanisms that contribute to larger horizontal inequities in DMP-CHD enrolment among women. Our study, conducted as proof of concept, underlines the importance of analysing equity in DMP enrolment in Germany as primary research focus using specific measures of SES beyond the routine national programme evaluation.

### Ethics statement

The ESTHER study was approved by the ethics committees of the medical faculty of the University of Heidelberg and of the medical board of the state of Saarland.

## Additional files

Additional file 1:
**Supplementary results and information on the model specification.**
Additional file 2:
**Estimating the Standard error of the Gini index.**
