# Supplementary material for: Analysing horizontal equity in enrolment in Disease Management Programmes for coronary heart disease in Germany 2008–2010
Source: Int J Equity Health. 2015 Mar 10;14:28. doi: 10.1186/s12939-015-0155-1 (PMC4357160; doi:10.1186/s12939-015-0155-1)
Supplement: Additional file 1: — Supplementary results and information on the model specification. [file 12939_2015_155_MOESM1_ESM.doc]

**Supplementary File 1**

**Analysing horizontal equity in utilisation of Disease Management Programmes for coronary heart disease in Germany 2008-2010**

Kayvan Bozorgmehr^§,1^ (MD, MSc), Miguel San Sebastian^2^ (MD, PhD), Hermann Brenner^3^ (MD, MPH), Oliver Razum^4^ (MD, MSc), Werner Maier^5^ (MPH), Kai-Uwe Saum^3^ (MPH), Bernd Holleczek^6^, Antje Miksch^1^ (MD), Joachim Szecsenyi^1^ (MD, MSc)

^§^ Corresponding author

1. Dept. of General Practice & Health Services Research, University Hospital Heidelberg, Heidelberg (Germany)
2. Division of Epidemiology and Global Health, Department of Public Health and Clinical Medicine, Umeå University, Umeå (Sweden)
3. Division of Clinical Epidemiology and Aging Research, German Cancer Research Center (DKFZ), Heidelberg (Germany)
4. Department of Epidemiology & International Public Health, School of Public Health, Bielefeld University, Bielefeld (Germany)
5. Institute of Health Economics and Health Care Management, Helmholtz Zentrum München - German Research Center for Environmental Health (GmbH), Neuherberg (Germany)
6. Saarland Cancer Registry, Saarbrücken (Germany)

Table S.1: Absolute frequency of enrolment and enrolment rates in the Disease Management Programme for coronary heart disease by educational attainment, regional deprivation and sex

|  |  | **Female** | | **Male** | | **Population** | |
| --- | --- | --- | --- | --- | --- | --- | --- |
|  |  | **Enrolled in DMP** | **Total** | **Enrolled in DMP** | **Total** | **Enrolled in DMP** | **Total** |
|  |  | n (%) | N (%) | n (%) | N (%) | n (%) | N (%) |
| **SES indicator** |  |  |  |  |  |  |  |
| **Highest**  **educational attainment** | I (lowest) | 89 (22.14) | 402 (100) | 204 (34.52) | 591 (100) | 293 (33.33) | 993 (100) |
|  | II | 14 (25) | 56 (100) | 28 (33.33) | 84 (100) | 42 (34.45) | 140 (100) |
|  | III (highest) | 3 (15) | 20 (100) | 49 (38.58) | 127 (100) | 52 (40) | 147 (100) |
|  | Column total | 106 (22.18) | 478 (100) | 281 (35.04) | 802 (100) | 387 (34.27) | 1280 (100) |
|  |  |  |  |  |  |  |  |
| **German Index of Multiple Deprivation** | Q1 (lowest SES/highest deprivation) | 27 (21.43) | 126 (100) | 63 (32.47) | 194 (100) | 90 (31.75) | 320 (100) |
|  | Q2 | 32 (19.51) | 164 (100) | 89 (36.03) | 247 (100) | 121 (32.84) | 411 (100) |
|  | Q3 | 18 (20.22) | 89 (100) | 50 (34.01) | 147 (100) | 68 (33.33) | 236 (100) |
|  | Q4 | 16 (25) | 64 (100) | 52 (36.62) | 142 (100) | 68 (37.95) | 206 (100) |
|  | Q5 (highest SES/lowest deprivation) | 12 (37.5) | 32 (100) | 24 (39.34) | 61 (100) | 36 (47.89) | 93 (100) |
|  | Column total | 105 (22.11) | 475 (100) | 278 (35.15) | 791 (100) | 383 (34.47) | 1266 (100) |
|  |  |  |  |  |  |  |  |

**SES**: socio-economic status. **Highest educational attainment**: Level I: no degree or minimum of nine years of education qualifying for professional training (Hauptschule). Level II: minimum of 10-11 years of education qualifying for professional training (Realschule/Mittlere Reife). Level III: minimum of 12-13 years of education qualifying for university entrance ('Fachhochschulreife/Abitur). **n (%)**: absolute number of persons enrolled in DMP and row percentages reflecting the utilization rate of DMPs within each stratum of respective SES indicators. **N**: total frequency of persons within each SES stratum including DMP participants and non-participants. **Q1-5**: quintiles of the German Index of Multiple Deprivration. Difference to N=1280 due to missing information on municipality ID (n=14). **DMP**: Disease Management Programme for coronary heart disease.

## Model specification

Due to the simultaneous clustering of patients in municipalities (patients’ residential area) and GP-practices (patients’ GP caring for their condition) our data showed a cross-classified structure, which means that conventional hierarchical multi-level models might not be directly applicable. In the cross-classified model, patients (level 1) are nested at level 2 in the cross-classification of “GPs-by-municipality” (or more specific: “GP-practices-by-patient-residential-area”).

We tested the model fit of cross-classified null-models (outcome: DMP-CHD enrolment) by means of likelihood-ratio tests (LR tests) against three alternative models. These were a single-level logistic regression model on the one hand, and “naive” two-level null-models on the other hand. In the latter, patients were nested either in (i) municipalities alone (Alternative 1) or (ii) GP practices alone (Alternative 2).

The test statistics of the LR test indicated that the unconditional cross-classified model was significantly preferred over a single-level logistic regression (chi^2^(2)= 19.69, p=0.0001), and over the “naive” two-level models with patients nested in municipalities (Alternative 1: chi^2^(1)= 6.15, p= 0.0132) or practices alone (Alternative 2: chi^2^(1)= 4.72, p= 0.0298).

Table S.2: Fixed and random effects of need and non-need variables on DMP-CHD enrolment obtained from a cross-classified multi-level logistic regression model (N=720)

|  | **Regression coef. (ß)** | **SE** | **z** | **p-value** | **[95% CI]** |
| --- | --- | --- | --- | --- | --- |
| ***Fixed effects*** | | | | | |
| CIRS G Severity Index | 0.49 | 0.2 | 2.49 | 0.01 | [0.1 ; 0.88] |
| SRH "fair/bad/very bad" (vs. "excellent/very good") | 0.08 | 0.17 | 0.45 | 0.65 | [-0.26 ; 0.42] |
| Age-group 65-74 (vs. 55-64) | 0.44 | 0.26 | 1.68 | 0.09 | [-0.07 ; 0.94] |
| Age-group 75-84 (vs. 55-64) | 0.54 | 0.27 | 1.98 | 0.05 | [0.004 ; 1.07] |
| Female (vs. Male) | 0.76 | 0.19 | 4.09 | <0.001 | [0.39 ; 1.12] |
| Immigration background (yes vs. no) | 0.43 | 0.3 | 1.42 | 0.16 | [-0.16 ; 1.02] |
| Social contacts ("2 and more" vs. "0-1") | 0.45 | 0.24 | 1.85 | 0.07 | [-0.03 ; 0.93] |
| Intercept | -2.53 | 0.51 | -4.97 | <0.001 | [-3.52 ; -1.53] |
| ***Random effects*** | | | | | |
|  | **Variance** | **SE** |  |  | **[95% CI]** |
| Municipality-level | 0.14 | 0.13 |  |  | [0.02 ; 0.82] |
| Practice-level | 0.24 | 0.19 |  |  | [0.05 ; 1.17] |
| ***Model sig.*** | | | | | |
| Wald-chi2 (df) |  |  | 28.74 (7) | 0.0002 |  |

SE: standard error. z: z statistic. CI: confidence interval. df: degrees of freedom. CIRS G: Cumulative Illness rating scale for geriatrics. SRH: self-rated health.

Table S.3: Fixed and random effects of need and non-need variables - including educational attainment - on DMP-CHD enrolment obtained from a cross-classified multi-level logistic regression model (N=720)

|  | **Regression coef. (ß)** | **SE** | **z** | **p-value** | **[95% CI]** |
| --- | --- | --- | --- | --- | --- |
| ***Fixed effects*** | | | | | |
| CIRS G Severity Index | 0.49 | 0.20 | 2.49 | 0.01 | [0.1 ; 0.88] |
| SRH "fair/bad/very bad" (vs. "excellent/very good") | 0.08 | 0.17 | 0.44 | 0.66 | [-0.27 ; 0.42] |
| Age-group 65-74 (vs. 55-64) | 0.43 | 0.26 | 1.68 | 0.09 | [-0.07 ; 0.94] |
| Age-group 75-84 (vs. 55-64) | 0.54 | 0.27 | 1.98 | 0.05 | [0.004 ; 1.07] |
| Female (vs. Male) | 0.76 | 0.19 | 4.06 | <0.001 | [0.39 ; 1.13] |
| Educational attainment ("level II + III" vs. "level I") | -0.02 | 0.20 | -0.09 | 0.93 | [-0.41 ; 0.37] |
| Immigration background (yes vs. no) | 0.43 | 0.30 | 1.42 | 0.16 | [-0.16 ; 1.03] |
| Social contacts ("2 and more" vs. "0-1") | 0.45 | 0.25 | 1.85 | 0.07 | [-0.03 ; 0.93] |
| Intercept | -2.52 | 0.51 | -4.96 | <0.001 | [-3.52 ; -1.53] |
| ***Random effects*** | | | | | |
|  | **Variance** | **SE** |  |  | **[95% CI]** |
|  |  |  |  |  |  |
| Municipality | 0.14 | 0.13 |  |  | [0.02 ; 0.82] |
| Practice | 0.24 | 0.19 |  |  | [0.05 ; 1.17] |
| ***Model sig.*** | | | | | |
| Wald-chi2 (df) |  |  | 28.74 (8) | 0.0004 |  |

SE: standard error. z: z statistic. CI: confidence interval. df: degrees of freedom. CIRS G: Cumulative Illness rating scale for geriatrics. SRH: self-rated health.
