# Supplementary material for: Analysing horizontal equity in enrolment in Disease Management Programmes for coronary heart disease in Germany 2008–2010
Source: Int J Equity Health. 2015 Mar 10;14:28. doi: 10.1186/s12939-015-0155-1 (PMC4357160; doi:10.1186/s12939-015-0155-1)
Supplement: Additional file 2: — Estimating the Standard error of the Gini index. [file 12939_2015_155_MOESM2_ESM.pdf]

Estimating the Standard error of the Gini index

Let the two vectors:

$$a(y) = \mu_y + y(2F(y) - 1) + \int_{-\infty}^{y} xF(x)dF(x) + y + 2\int_{y}^{\infty} (x - y)dF(x)dx$$

$$b(y) = 2\mu_y$$

Where the parameter μ_y denotes the average income (y), and F(x) is the CDF function. The Variance of the Gini index equals to the variance of the ratio: $E[a(y)]/\ E[b(y)]$ (See Duclos and Estban (2004)).

$$\text{Var(Gini)} = \text{Var}\ (u_a/\ u_b)$$

- Jean-Yves Duclos & Joan Esteban & Debraj Ray, 2004. "**Polarization: Concepts, Measurement, Estimation**," Econometrica, Econometric Society, vol. 72(6), pages 1737- 1772, November *(The formula can be found in this article).*

  http://onlinelibrary.wiley.com/doi/10.1111/j.1468-0262.2004.00552.x/pdf (equation10)

The STE of the concentration index is inspired from that of the Gini index, since the functional form of the Concentration index is quite similar to that the concentration index and the application y_i->t_i do not affect the structure of the sampling error (t is ranked based on y). Let the two vectors:

$$a(t(y)) = \mu_t + t(2F(y) - 1) + \int_{-\infty}^{y} t(y)F(x)dF(x) + t + 2\int_{y}^{\infty} (t(x) - t(y))dF(x)dx$$

$$b(t(y)) = 2\mu_t$$

$$\text{Var(Concentration)} = \text{Var}\ (u_a/\ u_b)$$
